# Unraveling Novel Strategies in Mesothelioma Treatments Using a Newly Synthetized Platinum(IV) Compound

**DOI:** 10.3390/pharmaceutics16081015

**Published:** 2024-07-31

**Authors:** Cristina Favaron, Ludovica Gaiaschi, Claudio Casali, Fabrizio De Luca, Federica Gola, Margherita Cavallo, Valeria Ramundo, Elisabetta Aldieri, Gloria Milanesi, Silvia Damiana Visonà, Mauro Ravera, Maria Grazia Bottone

**Affiliations:** 1 Department of Biology and Biotechnology, University of Pavia, Via Ferrata 9, 27100 Pavia, Italyludovica.gaiaschi@unipv.it (L.G.); claudio.casali@unipv.it (C.C.); fabrizio.deluca@unipv.it (F.D.L.); federica.gola@unipv.it (F.G.); margherita.cavallo01@universitadipavia.it (M.C.); gloria.milanesi@unipv.it (G.M.); 2 Department of Oncology, University of Torino, Via Santena 5/bis, 10126 Torino, Italy; valeria.ramundo@unito.it (V.R.); elisabetta.aldieri@unito.it (E.A.); 3 Unit of Legal Medicine and Forensic Sciences, Department of Public Health, Experimental and Forensic Medicine, University of Pavia, 27100 Pavia, Italy; silviadamiana.visona@unipv.it; 4 Department of Sciences and Technological Innovation (DiSIT), University of Piemonte Orientale “A. Avogadro”,Via Teresa Michel 11, 15121 Alessandria, Italy

**Keywords:** malignant mesothelioma, iron metabolism, ferroptosis, programmed cell death, platinum (IV) chemotherapeutics

## Abstract

Malignant mesothelioma is a rare tumor associated with asbestos exposure. Mesothelioma carcinogenesis is related to enhanced reactive oxygen species (ROS) production and iron overload. Despite the recent advances in biomedical sciences, to date the only available treatments include surgery in a small fraction of patients and platinum-based chemotherapy in combination with pemetrexed. In this view, the purpose of this study was to evaluate the therapeutic potential of the newly synthetized platinum prodrug Pt(IV)Ac-POA compared to cisplatin (CDDP) on human biphasic mesothelioma cell line MSTO-211H using different complementary techniques, such as flow-cytometry, transmission electron microscopy (TEM), and immunocytochemistry. Healthy mesothelial cell lines Met-5A were also employed to assess the cytotoxicity of the above-mentioned compounds. Our in vitro results showed that Pt(IV)Ac-POA significantly interfere with iron metabolisms and more importantly is able to trigger cell death, through different pathways, including ferroptosis, necroptosis, and apoptosis, in neoplastic cells. On the other hand, CDDP triggers mainly apoptotic and necrotic cell death. In conclusion, Pt(IV)Ac-POA may represent a new promising pharmacological agent in the treatment of malignant mesothelioma.

## 1. Introduction

Malignant pleural mesothelioma (MPM) is an extremely aggressive tumor with poor prognosis (generally the survival since the diagnosis is from 6 to 14 months) arising from the serosal linings of peritoneal, pleural, or pericardial cavity [1,2,3,4] and whose main risk factor is represented by asbestos exposure. Asbestos-induced carcinogenesis is mainly associated with iron overload and oxidative stress evoked by fibers, generating reactive oxygen species (ROS) and inducing mesothelial cells transformation [5,6]. Despite recent advances, treatment options for mesothelioma remain limited [1,2,7,8]. Currently, the only chemotherapeutic systemic treatment approved by the U.S. Food and Drug Administration (FDA) in 2004 for mesothelioma is combination chemotherapy based on cisplatin and pemetrexed. However, many MPM patients do not respond or progress during or after first-line treatment, leading to increased use of second-line therapies. Nevertheless, there is no definitive second-line treatment regimen for MPM. For patients who have undergone first-line platinum and pemetrexed-based therapy, retreatment with pemetrexed is suggested. Alternatively, if the same combination is used, premedication and desensitization strategies should be implemented [9].

After cancer development, malignant cells are continuously exposed to this pro-inflammatory microenvironment and become able to survive in an iron-rich environment, which promotes cell proliferation by accumulating DNA damages and consequently resulting in ferroptosis resistance [5,6]. Therefore, lowering iron levels and iron-induced ROS in cancer cells could represent a new strategy to overcome drug resistance.

On the other hand, iron exerts a fundamental role in asbestos bodies formation. Despite the fact that formation of asbestos bodies and their role in mesothelioma pathogenesis is still debated, it was suggested that their formation could have protective roles in people exposed to asbestos [10,11,12]. Furthermore, cisplatin, which is used in combination with pemetrexed in the standard first-line treatment for unresectable MPM, besides its well-known anticancer activity through DNA damage, can affect iron metabolism, leading to toxicity and cell death [13].

**Figure 1:**
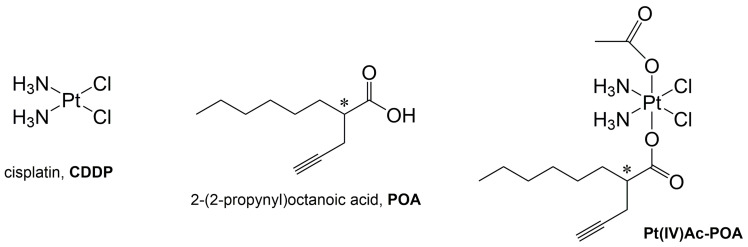
Structures of cisplatin and Pt(IV)Ac-POA. Sketch of cisplatin (CDDP), 2-(2-propynyl)octanoic acid, POA, and its Pt(IV) derivative (OC-6-44)-acetatodiamminedichlorido(2-(2-propynyl)octanoato)platinum(IV), Pt(IV)Ac-POA. The asterisk denotes a chiral center.

With the aim to combine multimodal strategy and perturbation of iron metabolism caused by platinum-based compounds, the (OC-6-44)-acetatodiamminedichlorido(2-(2-propynyl)octanoato)platinum(IV) complex, nicknamed Pt(IV)Ac-POA, is proposed here. This molecule is based on cisplatin and contains, as axial ligands, the 2-(2-propynyl)octanoic acid (POA), an MCFA-HDACi (medium-chain fatty acid histone deacetylase inhibitor) along with an inert acetate ligand (Ac) (Figure 1) [14]. Pt(IV) antitumor drug candidates are considered prodrugs since they can be reduced preferentially in the hypoxic (and then reducing) intracellular milieu of the tumor cells. If bioactive ligands are coordinated to the Pt(IV) core in the axial positions, the final bifunctional, hybrid, or multimodal anticancer prodrug will be able to deliver (almost selectively) two cytotoxic moieties (i.e., a Pt(II)-based metabolite as cisplatin, and the axial bioactive ligands), in the same place and at the same time, after in vivo reduction [15,16,17,18,19]. The possibility to co-administer cisplatin with Histone DeACetylase inhibitors (HDACi) seems to have a good effect on the increase of DNA-adducts formation [20,21,22].

Indeed, HDAC inhibition increases histone acetylation, decreasing histone–DNA interactions and allowing a higher sensitivity to DNA damage [23,24]. In addition, the combination between the cisplatin-like equatorial moiety and the MCFA produce a Pt(IV) complex, which is more lipophilic than cisplatin alone and shows promising antitumor activity both in vitro and in vivo on several human cancer cell lines with fewer side effects than cisplatin [15]. Recently, the interest for HDACi in mesothelioma management and other BAP1 mutated cancers, such as uveal melanoma, greatly increased, as demonstrated by several studies [25,26,27,28]. Finally, it was observed that Pt(IV)-POA was able to induce ferroptosis in iron-addicted cancer cells, such as human glioblastoma U251 cells [29,30].

In this study, Pt(IV)Ac-POA will be compared to the clinically used cisplatin, on human biphasic MSTO-211H mesothelioma cells and healthy human mesothelial Met-5a cells, with a particular attention on the alteration of iron metabolism and the activated cell death pathways, as well as morphological modifications.

## 2. Materials and Methods

### 2.1. Chemistry

The prodrug (OC-6-44)-acetatodiamminedichlorido(2-(2-propynyl)octanoato)platinum(IV) (Pt(IV)Ac-POA) was prepared according to established procedures [15]. Briefly, rac-2-(2-propynyl)octanoyl chloride (POA-Cl), obtained by reaction between rac-2-(2-propynyl)octanoic acid (POA) and oxalyl dichloride (1:5 molar ratio) in di-chloromethane, was reacted with (OC-6-44)-acetatodiamminedichloridohydroxidoplatinum(IV), obtained by oxidation of cisplatin with aqueous H_2_O_2_. The two compounds were mixed in a 1:5 (Pt:POACl) molar ratio in anhydrous acetone containing pyridine as a base (1:1 molar ratio vs. POACl) and left to react at reflux overnight. Then, the solvent was removed by rotary evaporation, and the crude residue was washed with hexane and water. Finally, the solid was dried under nitrogen flow to obtain Pt(IV)Ac-POA as a light-yellow powder with 84% yield.

### 2.2. Cell Culture and Treatments

Human biphasic mesothelioma MSTO-211H (ATTC CRL-2081, Rockville, MD, USA) cells and human mesothelial Met-5A cells (ATTC CRL-9444, Rockville, MD, USA) were cultured, respectively, in RPMI-1640 and M199 Medium (Euroclone, Milan, Italy) supplemented with 1% L-glutamine, 1% penicillin/streptomycin, and 10% fetal bovine serum (FBS) and maintained at 37 °C in a humidified atmosphere (95% air/5% CO_2_).

Forty-eight hours before the experiments, cells were seeded on glass coverslips (200,000 cells in 2 mL of media) for fluorescence microscopy or grown in 75 cm^2^ plastic flasks for flow cytometric and ultrastructural analysis at transmission electron microscope (TEM). Cells were treated for 12 h and 48 h at 37 °C of continuous treatments (CT) with the compounds under investigation, then flow cytometry, ultrastructural analysis, and immunocytochemistry analysis were performed. Concentrations of 10 μM for CDDP (Teva Pharma, Milan, Italy) and Pt(IV)Ac-POA were chosen after MTS Assay and reviewing the literature (Figures S1 and S2 and Table S1) [29,30,31,32,33].

### 2.3. Cell Cycle Analysis

Cells were washed in PBS (phosphate-buffered saline) for 30 s, permeabilized in 70% ethanol for 10 min, treated with RNase A 100 U mL^−1^ (Sigma-Aldrich, Milan, Italy), and then stained at room temperature with propidium iodide (PI) 50 μg mL^−1^ (Sigma-Aldrich, Milan, Italy) 1 h before flow cytometric analysis. PI red fluorescence was detected with a 610 nm long-pass emission filter. At least 10,000 cells per sample were measured to obtain the distribution among the different phases of the cell cycle and the percentage of dead cells. Data were collected using Cytometer BD FACS Lyric (Becton Dickinson, Franklin Lakes, NJ, USA) and analyzed with the built-in software (BD FACS Suite v1.3).

### 2.4. Transmission Electron Microscopy (TEM)

Control and treated MSTO-211H cells were harvested by mild trypsinization (0.25% trypsin in PBS containing 0.05% EDTA) and centrifugated at 800 rpm for 5 min in fresh tubes. The samples were immediately fixed with 2.5% glutaraldehyde in culture medium (2 h at room temperature) and washed several times with PBS. Samples were stained in 1% OsO4 for 2 h at room temperature (rt) and washed in distilled water. The cell pellets were pre-embedded in 2% agar, dehydrated with increasing concentrations of acetone (30%, 50%, 70%, 90%, and 100%), and finally were embedded in epoxy resin (Electron Microscopy Sciences, Hatfield, PA, USA, EM-bed812) and polymerized at 60 °C for 48 h. Ultrathin sections (60–80 nm) were cut on a Reichert OM-U3 ultramicrotome, collected on nickel grids, and stained with uranyl acetate and lead citrate. Lastly, sections were observed under a JEM 1200 EX II (JEOL, Peabody, MA, USA) electron microscope, equipped with a MegaView G2 CCD camera (Olympus OSIS, Tokyo, Japan) and operating at 100 kV.

### 2.5. Immunocytochemical Reactions: Fluorescence Microscopy Evaluation

Control and treated MSTO-211H and Met-5a cells were grown on glass coverslips, fixed with 4% formalin for 20 min, and post-fixed with 70% ethanol at −20 °C for at least 24 h. Samples were rehydrated for 10 min in PBS, and then unspecific sites were blocked by using a blocking solution of PBS supplemented with 4% BSA (bovine serum albumin) and 0.2% tween for 15 min at rt. Next, cells were immunolabeled with primary antibodies diluted in PBS-Tween 0.2% for 1 h, at rt in a dark moist chamber. After 3 washes in PBS of 5 min each, coverslips were incubated with secondary antibodies in PBS-Tween 0.2% (1:200, Alexa Fluor, Molecular Probes, Invitrogen, Waltham, MA, USA) for 45 min. At the end of the incubation and after other washing in PBS, sections were counterstained for DNA with 0.1 μg/mL Hoechst 33258 (Sigma-Aldrich, Milano, Italy), washed with PBS, and mounted in a drop of Mowiol (Calbiochem, Inalco, Italy) for fluorescence microscopy analysis. Primary and secondary antibodies used for immunocytochemical reactions at the fluorescence microscope are reported in Table 1. An Olympus BX51 microscope equipped with a 100 W mercury lamp was used under the following conditions: 330–385 nm excitation filter (excf), 400 nm dichroic mirror (dm) and 420 nm barrier filter (bf) for Hoechst 33258; 450–480 nm excf, 500 nm dm, and 515 nm bf for the fluorescence of Alexa 488; 540 nm excf, 580 nm dm, and 620 nm bf for Alexa 594. Images were recorded with an Olympus MagnaFire camera system and processed with the Olympus Cell F software version 3.1 (Olympus Italia Srl, Segrate, MI, Italy). To make the fluorescence intensity comparable, during image acquisition, the exposure time to detect every single fluorescence was selected based on the control sample and then maintained constant for the respective experimental conditions, thus avoiding the insertion of any variables in the analysis. The fluorescence intensity of the proteins of interest was analyzed with the CellProfiler software version 4.2.6 [34].

### 2.6. Statistical Analysis

For immunofluorescence quantification, at least 100 cells from 11 quadrants were evaluated for a random analysis, and the values obtained were expressed as mean ± SEM (standard error of the mean) in arbitrary fluorescence unit (a.f.u.) and normalized to control conditions. One-way ANOVA and post hoc Dunnet’s test were used to analyze data differences for Ki-67 and acetyl-H3 fluorescent staining in mesothelioma cells. Data differences between MSTO-211H and Met-5a cell lines were analyzed for statistical significance using two-way ANOVA and post hoc Turkey’s test (software package GraphPad Prism Inc., version 10.2.3, La Jolla, CA, USA). *p*-values < 0.05 were considered statistically significant.

## 3. Results

### 3.1. Cell Cycle Analysis in MSTO-211H Cell Line

**Figure 2:**
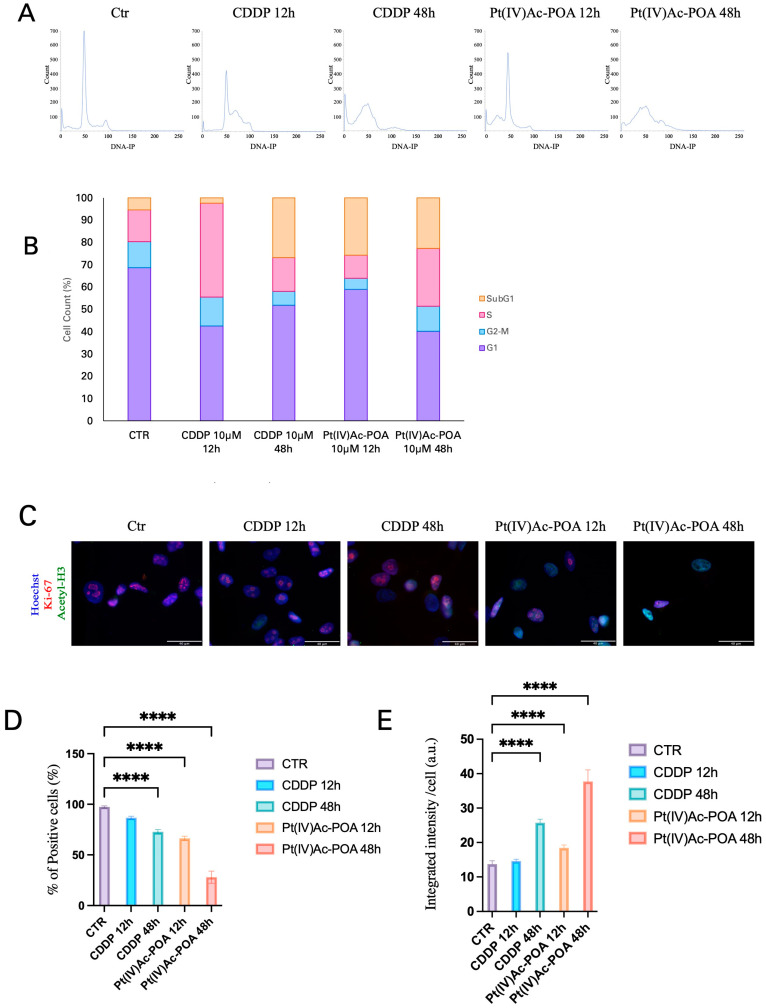
CDDP and Pt(IV)Ac-POA affect cell cycle and histone acetylation. (**A**) Cytofluorimetric analysis. Graphs showing DNA content after PI staining human MSTO-211H cells in control condition and after 12 h and 48 h of CT with CDDP and Pt(IV)Ac-POA treatments (10μM). (**B**) Stacked histograms displaying the percentage of events recorded across the different cell cycle phases after treatments. (**C**) Control samples vs. treated cells after 12 h and 48 h of CT to CDDP and Pt(IV)Ac-POA (10 μM). Double immunofluorescence reaction, with the relative quantification, for Ki-67 (red) and acetyl-H3 (green), nuclei were counterstained with Hoechst 33258 (blue) Bar = 40 μm; magnification: 60×. (**D**,**E**) Histograms illustrating the results of imaging and statistical analysis for (**D**) Ki-67 positive cells and (**E**) Acetyl-H3 fluorescence quantification. One-way ANOVA test: *p* < 0.0001. * statistical significance respect to control condition is marked by an asterisk; *p*-values: (****) *p* < 0.0001.

To investigate the effect of treatments with CDDP and Pt(IV)Ac-POA on the cell cycle, DNA was stained with IP and analyzed using flow cytometry in mesothelioma cells. As reported in Figure 2A,B, CDDP- and Pt(IV)Ac-POA-treated samples showed strong alterations in terms of DNA content distribution. After 12 h of CT with CDDP, cells in G2-S phases tend to accumulate, while after 12 h and 48 h of Pt(IV)Ac-POA exposure, cells in S and G2 strongly decreased. Moreover, a sub-G1 peak was clearly present only after treatments with Pt(IV)Ac-POA. Conversely, after 48 h of CT with both CDDP and Pt(IV)Ac-POA, the G1, S, and G2 peaks were totally absent with the exception of a small G2 peak detected in CDDP treated samples.

Ki-67 and acetyl-H3 expression was then evaluated by double immunofluorescence reaction after treatments with CDDP and Pt(IV)Ac-POA (Figure 2C). The amount of Ki-67-positive cells was significantly lower compared to the control condition after 48 h of exposure to CDDP (*p* < 0.0001) and after both 12 and 48 h of treatment with Pt(IV)Ac-POA (*p* < 0.0001 and *p* < 0.0001, respectively) (Figure 2D).

As reported in Figure 2E, the acetyl-H3 fluorescence intensity was significantly higher after 48 h of CT (*p* < 0.0001). Notably, the acetylation of H3 was significantly stronger after treatment with Pt(IV)Ac-POA compared to CDDP (*p* < 0.0001).

### 3.2. Ultrastructural Analysis

**Figure 3:**
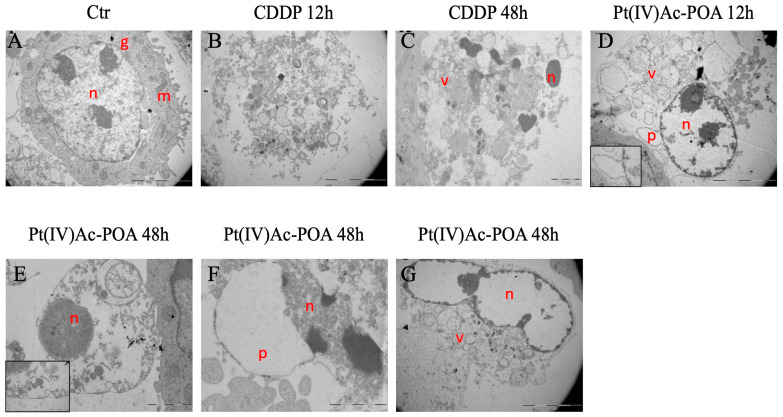
Ultrastructural analysis reveals the activation of different pathways of cell death. (**A**) MSTO-211H cells in control conditions. (**B**,**C**) Cells treated for 12 and 48 h with CDDP 10 μM. Examples of necrosis (**B**) and apoptosis (**C**). (**D**–**G**) Cells after 12 and 48 h of CT with 10 μM -Pt(IV)Ac-POA prodrug. Examples of (**D**–**F**) necroptosis, (**E**) apoptosis with some vesicles enclosing cell debris (insert), and (**G**) ferroptosis. Legend: nucleus (n), Golgi apparatus (g), mitochondria (m), citoplasmic vesicles (v), perinuclear space (p).

In order to better understand the effects of the studied compounds, morphological changes in MSTO-211H cells were analyzed by transmission electron microscopy. Untreated cells (Figure 3A) were characterized by the nucleus in a central position (indicated by the letter “n”), a decondensed chromatin, and a well-organized Golgi apparatus (indicated by the letter “g”) in the perinuclear zone. While in CDDP-treated samples necrosis (Figure 3B) and apoptosis (Figure 3C) were observed, in Pt(IV)Ac-POA samples we detected both apoptosis (Figure 3E) and necroptosis (Figure 3D,F). The latter is characterized by both apoptotic features (i.e., karyorrhexis, indicated by the letter “n”), and necrotic ones (i.e., cytoplasmic vacuolations, indicated by “v” in Figure 3D and plasma membrane rupture). Enlargement of perinuclear spaces (indicated by letter “p” in Figure 3D–F) and nuclear envelope detachment were also observed (insert Figure 3E). After 48 h of continuous treatment with Pt(IV)Ac-POA, cells with an extreme chromatin decondensation (letter ”n”), a destroyed mitochondria cristae, and cytoplasm charged of vesicles (letter “v”) were observed, indicating the presence of ferroptotic cells (Figure 3G).

### 3.3. BAP1 and NF2 Modulation

**Figure 4:**
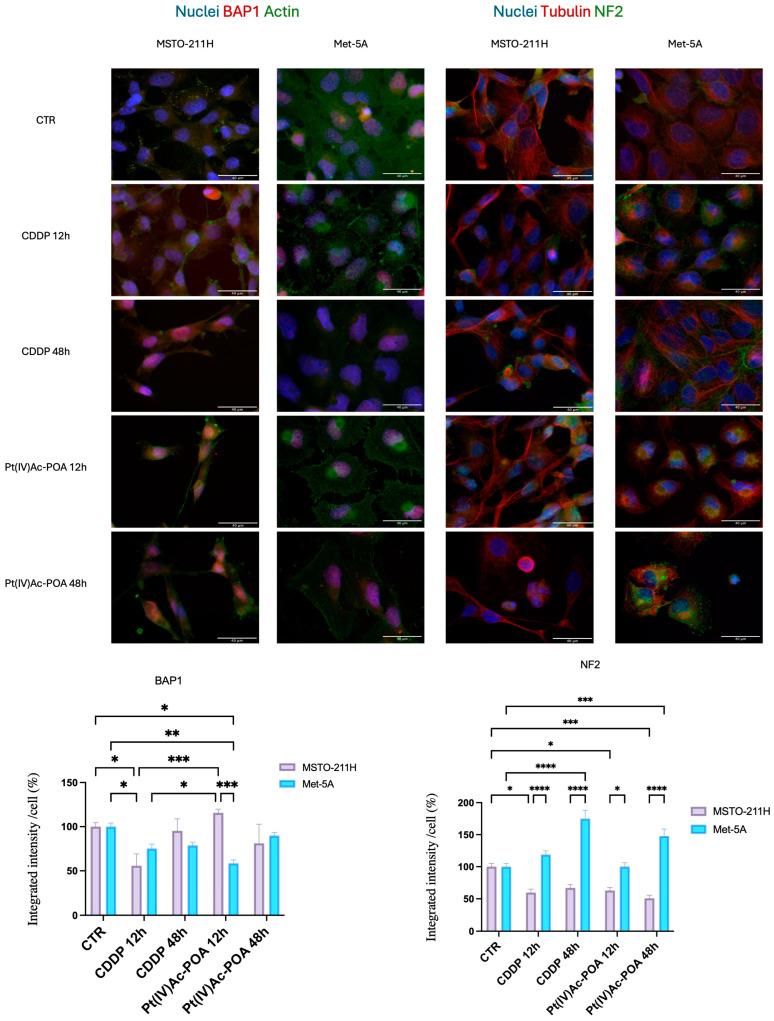
BAP1 and NF2 expression are altered by the pharmacological insults. Double immunocytochemical reaction for BAP1 (red fluorescence)*—*α-tubulin (red) and NF2 (green fluorescence)*—*β actin (green); DNA counterstaining with Hoechst 33258 (blue fluorescence) in control condition and CDDP- and Pt(IV)Ac-POA (10 μM)-treated samples (both MSTO-211H and Met-5A cells). Bar = 40 μm; magnification: 60×. Histogram represents the relative expression of BAP1 and NF2 for MSTO-211H (pink) and Met-5A (blue) cell lines. Statistical analysis: Two-way ANOVA test: *p* < 0.0001. * statistical significance with respect to control condition is marked by an asterisk; *p*-values: (*) *p* < 0.05, (**) *p* < 0.01, (***) *p* < 0.005, (****) *p* < 0.0001.

Since BAP1 (BRCA1-Associated Protein 1) and NF2 (Neurofibromin 2) are frequently mutated in mesothelioma and exploit fundamental functions in regulating cell death, proliferation, and chromatin organization [35,36,37,38,39], their expression was evaluated by immunofluorescence in MSTO-211H and Met-5a cells. As expected, under the control condition and in both cell lines, the expression of NF2 and BAP1 was maintained Figure 4) while the cytoskeleton was well structured in actin and tubulin filaments within all cytoplasm. In mesothelioma cells, NF2 expression was found to be significantly downregulated in all tested conditions apart from 12 h of CT with Pt(IV)Ac-POA, and the signal was localized mainly in the cytoplasm (Figure 4). Conversely, in normal cell lines, NF2 expression was found to be significantly upregulated in respect to the control condition after long-lasting treatment with both compounds (the results of the statistical analysis are shown in Figure 4).

BAP1 signal localized mainly in the nucleus (Figure 4), and its expression was significantly higher in CDDP- and Pt(IV)Ac-POA-treated mesothelioma cells at 12 h compared to untreated mesothelioma cells (*p* < 0.0001). On the other hand, Met-5a cells displayed again an opposite trend, even though it was statistically significant only after the longer lasting treatments with cisplatin with respect to the negative control.

### 3.4. Impairment of Iron Metabolisms and Ferroptotic Pathway

Next, oxidative stress, iron metabolism, and ferroptotic pathways were evaluated by immunofluorescence.

**Figure 5:**
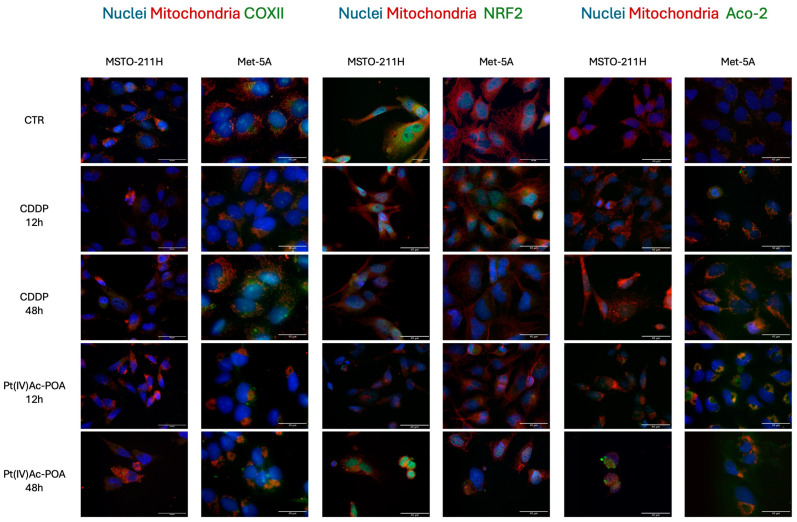
Pt(IV)Ac-POA enhances ROS production. Double immunocytochemical reaction for COX2 (green fluorescence), NRF2 (green fluorescence), Aconitase-2 (green fluorescence), and mitochondria (red fluorescence); DNA counterstaining with Hoechst 33258 (blue fluorescence) in control condition and CDDP and Pt(IV)Ac-POA (10 μM) in MSTO-211H and Met-5A cell lines Bar = 40 μm; magnification: 60×.

Firstly, COX2 (Cyclooxygenase 2) expression was investigated since both CDDP and Pt(IV)Ac-POA can result in the production of ROS (Figure 5). Considering both cell lines, a significant downregulation of COXI2 was observed across all the tested conditions, apart from 48 h of CT with CDDP and 48 h of Pt(IV)Ac-POA exposure in Met-5a cells (Figure 5). Consequently, other ROS responsive elements, namely NRF2 (NF-E2-related factor 2) and Aco2 (Aconitase 2), also related to the ferroptotic pathways were investigated. While NRF2 signal was found to be significantly higher in Met-5a cells, mitochondrial aconitase-2 was upregulated in MSTO-211H cells (Figure 5).

**Figure 6:**
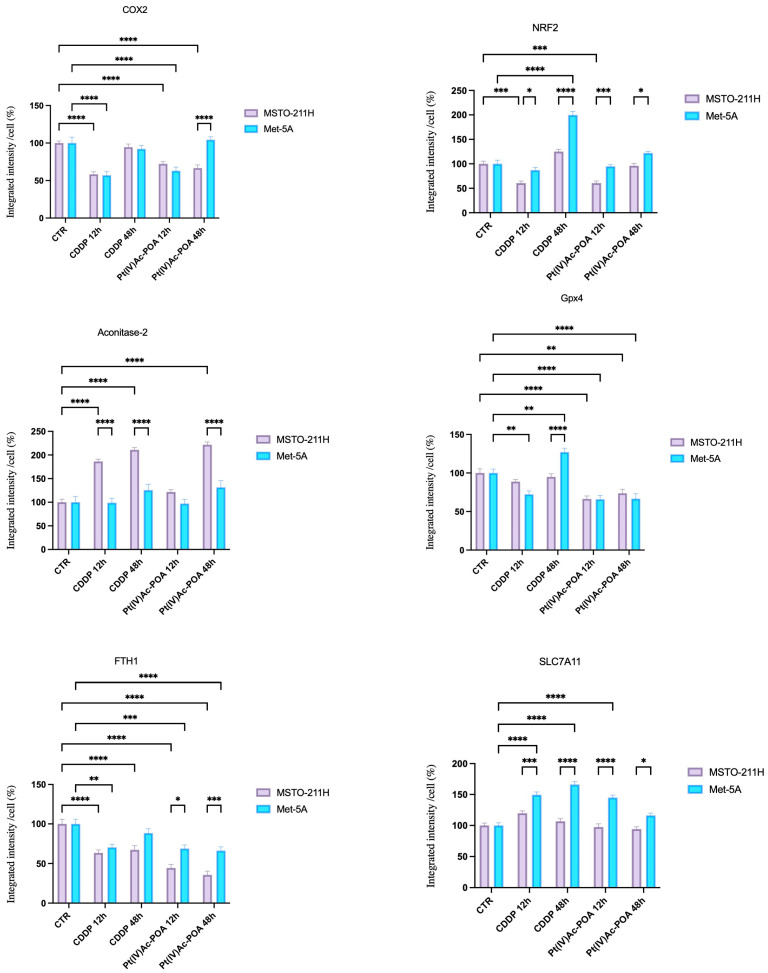
Fluorescent quantification of the investigated markers. Histogram represents the relative expression of COX2, NRF2, Aco2, GPX4, FTH1, and SLC7A11 in the control condition, CDDP, and Pt(IV)Ac-POA (10 μM). Statistical analysis: Two-way ANOVA test. * statistical significance with respect to control condition is marked by an asterisk; *p*-values: (*) *p* < 0.05, (**) *p* < 0.01, (***) *p* < 0.005, (****) *p* < 0.0001.

**Figure 7:**
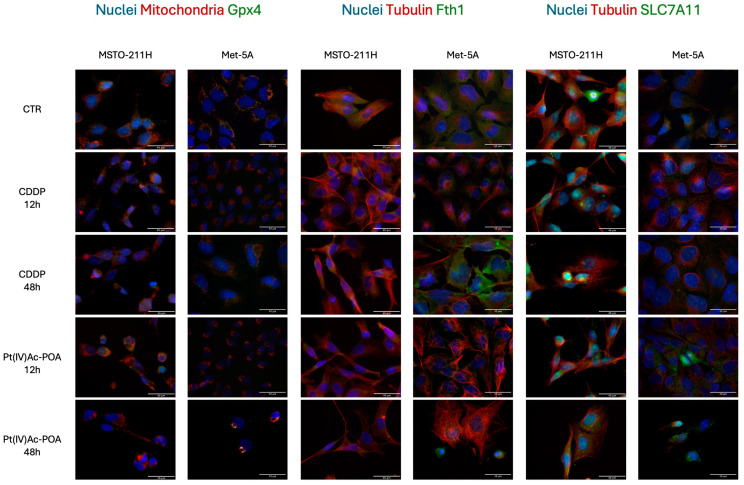
Pt(IV)Ac-POA triggers the ferroptotic pathway. Double immunocytochemical reaction for GPX4 (green fluorescence)—mitochondria (red fluorescence), FTH1 (green fluorescence)*—*α-tubulin (red fluorescence), and SLC7A11 (green fluorescence)*—*α-tubulin (red fluorescence); DNA counterstaining with Hoechst 33258 (blue fluorescence) in control condition and CDDP and Pt(IV)Ac-POA (10 μM). Bar = 40 μm; magnification: 60×.

Finally, the relative expression of ferroptosis indicators was assessed by immunofluorescence analysis. As illustrated in Figure 6, in the control condition, cells exhibit a mild immunopositivity for GPX4 (Glutathione peroxidase 4) that colocalized with mitochondria, which showed their characteristic spotted-like shape. Moving to samples treated with platinum-based compounds, as shown in Figure 6, we observed a significant decrease in GPX4 expression after 12 and 48 h of CT of Pt(IV)Ac-POA in both cell lines (*p*-values are illustrated in Figure 6). In particular, cells lost their morphology, and mitochondria distribution was severely impaired, with formation of clusters around the nucleus, especially in cells that showed apoptotic appearance (Figure 7).

Moreover, FTH1 (Ferritin Heavy chain 1) expression was severely altered in all CDDP- and Pt(IV)Ac-POA-treated cells (*p* < 0.0001), thereby suggesting a severe iron metabolism impairment (Figure 6 and Figure 7). However, MSTO-211H cells treated for 48 h with Pt(IV)Ac-POA also exhibited significantly higher levels of a ferroptosis inhibitor Aconitase-2 (Figure 6 and Figure 7) in respect to untreated samples (*p* < 0.001). SLC7A11 (cystine transporter Solute Carrier family 7 member 11) was found to be significantly upregulated only in Met-5a cells in all the tested conditions, with the exception of the longer timepoints of Pt(IV)Ac-POA. The ANOVA and post hoc comparisons are shown in Figure 6.

### 3.5. Activation of Different Cell Death Mechanisms

**Figure 8:**
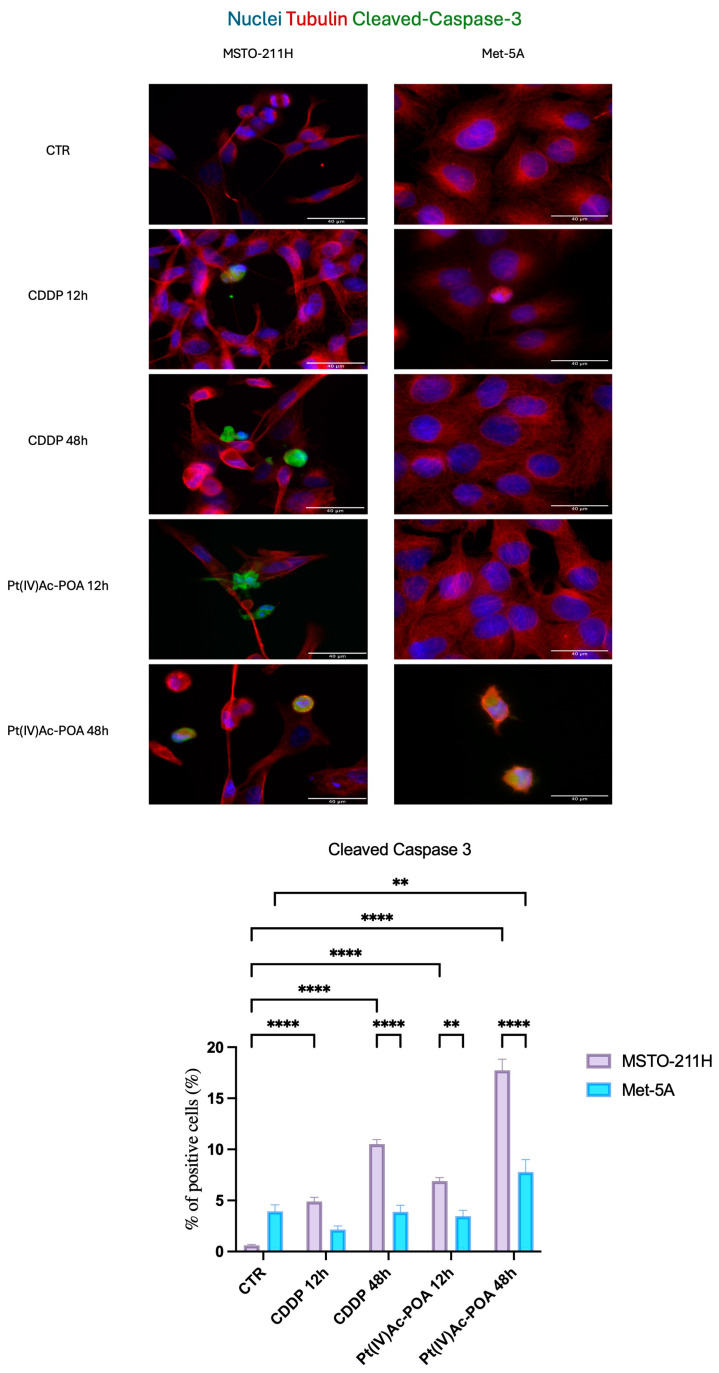
Cleaved caspase-3 is detected after treatments in MSTO-211H cell line. Double immunocytochemical reaction for cleaved caspase-3 (green fluorescence) and α-tubulin (red fluorescence); DNA counterstaining with Hoechst 33258 (blue fluorescence) in control condition, CDDP-, and Pt(IV)Ac-POA-treated samples (10 μM). Bar = 40 μm; magnification: 60×. Histogram showing the percentage (%) of positive cleaved caspase-3 cells. Statistical analysis: Two-way ANOVA test. * statistical significance with respect to control condition is marked by an asterisk; *p*-values: (**) *p* < 0.01, (****) *p* < 0.0001.

Finally, to better understand the results obtained by transmission electron microscope, other cell death mechanisms were evaluated. First, to analyze the activation of the intrinsic apoptotic pathway, immunocytochemical reaction to cleaved caspase-3 was conducted. As shown in Figure 8, in the control conditions of both cell lines, no expression of active caspase-3 was detected, consistently with the presence of only viable cells. The actin cytoskeleton was well structured in filaments within all cytoplasm. After treatments with both platinum-based compounds, MSTO-211H cells underwent apoptosis; the percentage of immuno-positive cleaved caspase-3 cells was significantly increased, and cell morphology was altered: cells developed a round shape and their nuclei appeared fragmented. The actin cytoskeleton collapsed, with inhomogeneous distribution localized around the nucleus (Figure 8). Conversely, only the longer lasting Pt(IV)Ac-POA treatment triggered the intrinsic apoptotic pathway in Met-5a cells (*p* < 0.0001). Of note, the percentage of cleaved caspase-3-positive mesothelioma cells was significantly higher after 48 h of CDDP and Pt(IV)Ac-POA exposures compared to the healthy cells (Figure 8).

Autophagy and necroptosis were also evaluated by IF.

**Figure 9:**
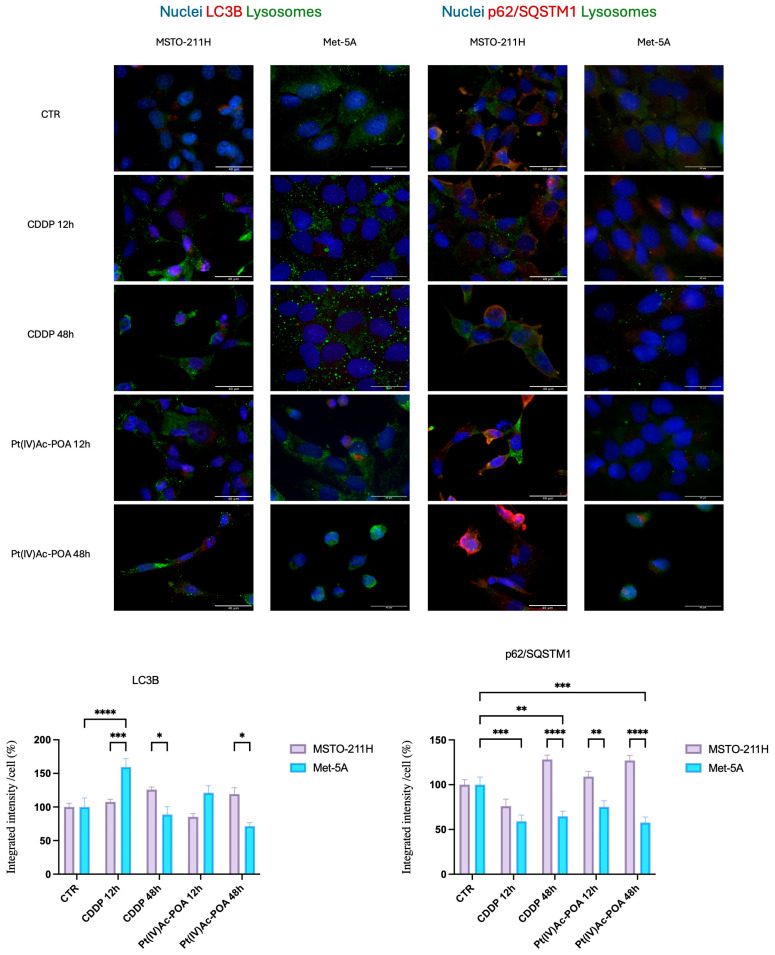
Pt(IV)Ac-POA causes the blockage of the autophagic flux in MSTO-211H cells. Double immunocytochemical reaction for LC3B (red fluorescence), p62/SQSTM1 (red fluorescence), and lysosomes (green fluorescence); DNA counterstaining with Hoechst 33258 (blue fluorescence) in control condition, CDDP-, and Pt(IV)Ac-POA0-treated samples (10 μM). Bar = 40 μm; magnification: 60×. Histogram showing the fluorescence quantification for LC3B and p62/SQSTM1. Statistical analysis: Two-way ANOVA test. * statistical significance with respect to control condition is marked by an asterisk; *p*-values: (*) *p* < 0.05, (**) *p* < 0.01, (***) *p* < 0.005, (****) *p* < 0.0001.

In the control conditions, LC3B (Microtubule-associated proteins light chain 3B) and p62/SQSTM1 (Sequestosome 1) are localized in the nucleus and cytoplasm within lysosomes (Figure 9). However, the LC3B fluorescent signal was found to be dysregulated only in Met-5A cells, after CDDP exposure and the longest Pt(IV)Ac-POA treatment. In contrast, the p62/SQSTM1 signal was found significantly higher in MSTO-211H cell line, especially after 48 h of CT with both platinum-based compounds. Met-5A displayed an opposite trend, reaching statistical significance after 48 h of both CDDP and Pt(IV)Ac-POA exposures (Figure 9).

**Figure 10:**
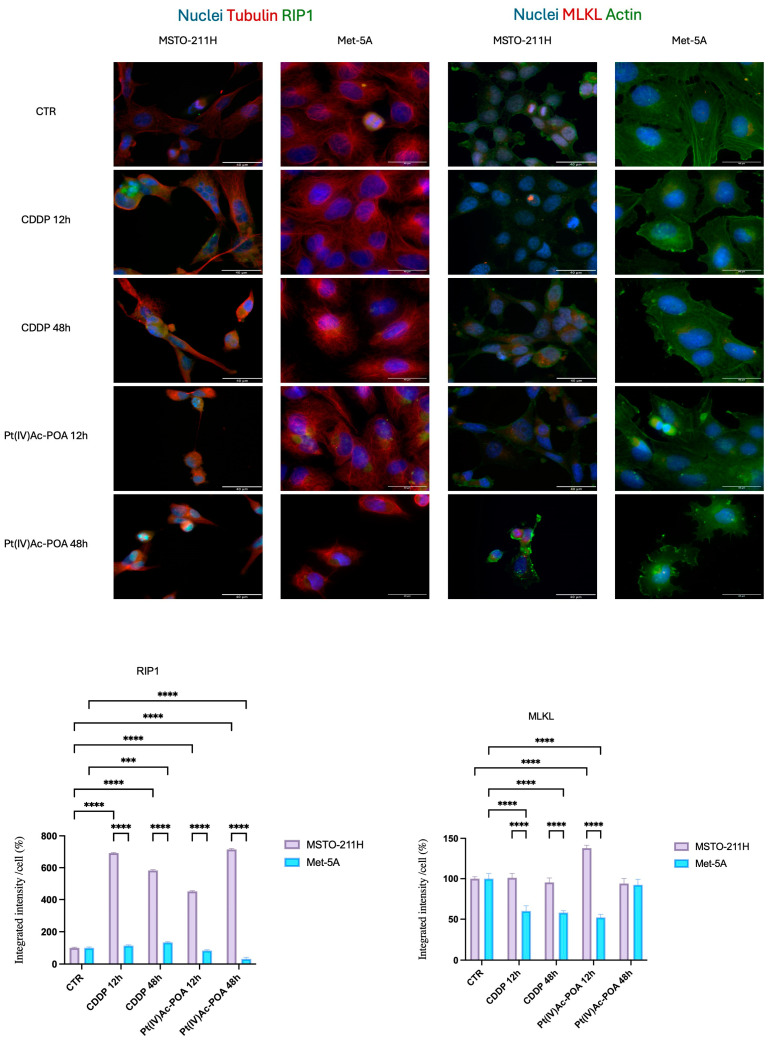
Pt(IV)Ac-POA and CDDP trigger necroptotic cell death only in mesothelioma cell lines. Double immunocytochemical reaction for RIP1 (green fluorescence)—α-tubulin (red fluorescence) and MLKL (red fluorescence)—actin (green fluorescence); DNA counterstaining with Hoechst 33258 (blue fluorescence) in control condition, CDDP-, and Pt(IV)Ac-POA-treated samples (10 μM). Bar = 40 μm; magnification: 60×. Histogram showing the fluorescence quantification for RIP1 and MLKL. Statistical analysis: Two-way ANOVA test. * statistical significance with respect to control condition is marked by an asterisk; *p*-values: (***) *p* < 0.005, (****) *p* < 0.0001.

RIP1 (Receptor-Interacting Protein 1) immunolabelling showed that in control conditions, contrary to what was observed in the samples subjected to CT, the fluorescent signal was diffused homogeneously along the entire cytoplasm and was not revealed in or around the nucleus (Figure 10).

Even in CDDP and Pt(IV)Ac-POA treated MSTO-211H samples, RIP1 was found significantly upregulated (*p* < 0.0001). MLKL (Mixed Lineage Kinase domain-Like protein) seemed to localize mainly in perinuclear spots in all the samples; its expression was significantly upregulated only after 48 h of Pt(IV)Ac-POA treatment (*p* < 0.0001), while in the others conditions the signal levels remained similar to the controls. Conversely, a general downregulation of MLKL has been detected in Met-5a cells, regardless of the experimental condition (Figure 10).

## 4. Discussion

Despite the recent advances in biomedical research, the only approved mesothelioma treatments include cisplatin–pemetrexed doublet and immunotherapy combination with Nivolumab and Ipilimumab [40]. However, platinum-based drugs are associated with heavy side effects and the onset of drug resistance mechanisms. Furthermore, it is already noted that the induction of oxidative stress and hypoxia due to cisplatin treatment, as well as tobacco smoke exposure, could result in cancer development, progression, and the onset of drug resistance [41,42]. Novel strategies to overcome pharmacological resistance of cancer cells is represented by the usage of HDACi, as explained before [20,21,22]. Accordingly with our previous studies, the most marked effects have been observed after Pt(IV)Ac-POA treatments [14,29,30,33]. Namely, after treatment with this compound, we observed high cell mortality and lower proliferation. Further experiments revealed that several cell death pathways were altered: apoptosis, ferroptosis, and autophagy. As expected, CDDP exerted much lower cytotoxic effects in terms of cell mortality. The higher cytotoxicity of Pt(IV)Ac-POA compared to cisplatin was confirmed, in both flow-cytometry and ultrastructural microscopy analysis. More precisely, the association of the HDAC inhibitory molecule on the cisplatin sketch resulted in a more decondensed chromatin that enhanced the antitumor effects of the alkylating core. With the ultrastructural analysis, we confirmed as well that the newly synthetized prodrug activated multiple mechanisms of cell death, thus confirming our previous studies on glioblastoma cells [29,30,33]. Importantly, while cisplatin triggered necrotic cell death, after Pt(IV)Ac-POA exposure, more apoptotic, necroptotic, and ferroptotic cells were detected. This means that Pt(IV)Ac-POA preferentially induces a regulated cell death and, therefore, it is characterized by a lower systemic toxicity for the human organism. This result is of particular importance since the currently available therapies, based on platinum compounds, provoke devastating side effects on the patients due to their high toxicity; thus, Pt(IV)Ac-POA might represent a step toward a more tolerable mesothelioma therapy.

To better characterize the properties of the tested pharmacological treatments, here we investigated their effect on ferroptosis, given its importance in mesothelioma. In ferroptosis, cell death is executed by ROS-mediated oxidation of polyunsaturated fatty acids (PUFAs). Lipid peroxidation is prevented by glutathione peroxidase 4 (GPX4), which uses reduce glutathione (GSH), synthetized from cystine, as reducing agent [43]. BAP1 suppresses SLC7A11, a cystine transporter, while NF2 prevents LATS2 accumulation into the nucleus thereby inhibiting the transcription ASLC4, a ferroptosis promoter [43,44]

In the present work, we demonstrated that, after treatments with the new prodrug, significantly lower levels of GPX4 and FTH1 were detected in mesothelioma cells, indicating the activation of ferroptosis, supporting the ultrastructural analysis.

Next, we investigated whether the tested compounds were able to induce other forms of cell death, such as apoptosis and necroptosis by immunofluorescence on both cell lines. It is known that enhanced ROS production could trigger apoptosis by disrupting the mitochondrial membrane, phenomena observed during our ultrastructural analysis; immunocytochemical staining indeed confirmed the activation of caspase-3 in CDDP- and Pt(IV)Ac-POA-treated samples as well as a significant downregulation of COX2, especially in mesothelioma cells. Importantly, the downregulation of COX2 pointed to a general ROS accumulation, especially after both 12 h and 48 h of CT with Pt(IV)Ac-POA, therefore explaining the higher cytotoxicity of the new prodrug compared to CDDP. Of note, the percentage of caspase-3-positive cells after Pt(IV)Ac-POA exposure in mesothelial cells was similar to that observed in controls. At the same time, the percentage of apoptotic Met-5a cells was significantly lower compared to what was observed in mesothelioma cells. Taken together, these data suggested that the new prodrug selectively kills mesothelioma cells.

Necroptosis is another form of programmed cell death executed through the formation of necrosome, which requires RIP1, RIP3, and MLKL. Specifically, RIP1 recruits RIP3, which becomes phosphorylated and active. At this point, the necrosome is formed and RIP3 recruits and phosphorylates MLKL, which undergoes conformational changes, translocating to plasma membrane and to the nucleus, causing their permeabilization and finally cell death [45,46]. Our experiments revealed an MLKL fluorescence signal in the perinuclear region of cells after exposure to Pt(IV)Ac-POA. In contrast, no necroptosis was observed among CDDP MSTO-211H cells. In addition, while MLKL was found downregulated after 12 h and 48 h of CDDP treatment, we observed a significantly higher signal of MLKL after 12 h of CT with Pt(IV)Ac-POA. This supports the idea that necroptosis can be regarded as a possible therapeutic target in mesothelioma therapy with the compounds tested here. Importantly, no significant alterations of the MLKL signal pointing to the activation of necroptotic pathway have been detected in Met-5A cells.

In the present study, the autophagic pathway was also investigated through the expression of its main effectors, LC3B and p62/SQSTM1. After 48 h of CT with Pt(IV)Ac-POA cells, both LC3B and p62/SQSTM1 significantly increased compared to control conditions. In contrast, in CDDP-exposed cells, the two markers displayed opposite trends, namely an increment for LC3B and a decrement for p62/SQSTM1 (even if both did not reach the statistical significance). These differences were particularly interesting, since they allowed us to distinguish between the activation of the autophagic pathways and the autophagic flux blockage. In physiological conditions, during the activation of autophagy, LC3B is inserted into the autophagolysosomes’ membranes, while p62/SQSTM1 is recruited later, when fusion with lysosomes occurs and damaged organelles are degraded [47,48]. The promotion of autophagy has been linked to chemoresistance: cells take advantage by using the damaged organelles to survive in stressful conditions [49]. On the other hand, when both LC3B and p62/SQSTM1 levels augmentation occur, autophagolysosomes are formed but not degraded. This blockage of the autophagic flux resulted in vesicle accumulation that finally could result in enhanced cell death through other mechanisms, such as apoptosis, ferroptosis, necroptosis, and reducing the survival of damaged cells [48,50,51]. Therefore, we can speculate that autophagy blockage can be a promising therapeutic strategy and that Pt(IV)Ac-POA cells showed effects in such a direction.

Overall, as also supported by preliminary in vivo results [15], we can speculate that this new prodrug could be a safer option, with less cytotoxic effects compared to the widely used cisplatin. This is a very important result since the onset of severe side effects as well as the establishment of drug resistance mechanisms greatly affect therapy outcomes. Importantly, considering the healthy cells tested here, we did not observe major impairments of the investigated cell death pathways after Pt(IV)Ac-POA treatments, suggesting a better tolerability of this compound. However, further studies to better elucidate the mechanisms of action of this compound, including its ability to act as histone acetylase inhibitors and its interplay with pemetrexed, should be performed. Additionally, it will be desirable to confirm these results in different cell lines mutated or silenced for BAP1, NF,2 and CDKN2A and in in vivo models and in to assess the impact of those genes on the above-mentioned pathways and to better understand potential short- and long-term side effects of the new compound.

## Figures and Tables

**Table 1:** Primary and secondary antibodies used for immunofluorescence reactions.

Primary Antibody		Dilution	Secondary Antibody
BAP1	Mouse monoclonal anti-BAP1 (Santa Cruz Biotechnology, Dallas, TX, USA)	1:200	Alexa 594-conjugated anti-mouse (Molecular Probes; Invitrogen, Waltham, MA, USA)
GPX4	Rabbit polyclonal anti-Glutathione Peroxidase 4 (GPX4) (Abcam, Cambridge, UK)	1:400	Alexa 488-conjugated anti-rabbit (Molecular Probes; Invitrogen)
FTH1	Rabbit anti-ferritin heavy chain (FTH1) (Invitrogen)	1:200	Alexa 488-conjugated anti-rabbit antibody (Molecular Probes; Invitrogen)
NF2 antibody	Rabbit polyclonal anti-NF2 (Sigma-Aldrich, St. Louis, MO, USA)	1:100	Alexa 488-conjugated anti-rabbit (Molecular Probes; Invitrogen)
SLC7A11	Mouse polyclonal anti-SLC7A11 (Invitrogen)	1:100	Alexa 594-conjugated anti-mouse (Molecular Probes; Invitrogen)
Cleaved Caspase 3	Rabbit monoclonal anti-cleaved caspase-3 (Cell Signaling Technology, Danvers, MA, USA)	1:200	Alexa 488-conjugated anti-rabbit (Molecular Probes; Invitrogen)
RIP1	Rabbit polyclonal anti-RIP1 (Santa Cruz Biotechnology)	1:200	Alexa 488-conjugated anti-rabbit (Molecular Probes; Invitrogen)
MLKL	Mouse monoclonal Anti-MLKL Antibody, clone 3H1 (Sigma-Aldrich)	1:200	Alexa 594-conjugated anti-mouse (Molecular Probes; Invitrogen)
LC3B	Rabbit polyclonal anti-LC3B (Cell Signaling Technology)	1:200	Alexa 594-conjugated anti-rabbit (Molecular Probes; Invitrogen)
p62/SQSTM1	Mouse monoclonal anti-mouse p62/SQSTM1 (Abcam)	1:200	Alexa 594-conjugated anti-mouse (Molecular Probes; Invitrogen)
ACO-2	Rabbit polyclonal anti-aconitase-2 (Aco-2) (GeneText, Irvine, CA, USA)	1:200	Alexa 488-conjugated anti-rabbit (Molecular Probes; Invitrogen)
NRF2	Rabbit polyclonal anti-NRF2 (Abcam)	1:200	Alexa 488-conjugated anti-rabbit (Molecular Probes; Invitrogen)
Ki-67	Mouse monoclonal anti-Ki-67 antibody (Dako, Agilent technologies, Santa Clara, CA, USA)	1:100	Alexa 594-conjugated anti-mouse (Molecular Probes; Invitrogen)
ACETYL-H3	Rabbit polyclonal anti-Histone H3 (acetyl K9) (Abcam)	1:200	Alexa 488-conjugated anti-rabbit (Molecular Probes; Invitrogen)
β-Actin	Rabbit polyclonal anti-beta actin (GeneText)	1:200	Alexa 488-conjugated anti-rabbit (Molecular Probes; Invitrogen)
α-Tubulin	Monoclonal mouse anti-α-tubulin (Invitrogen)	1:1000	Alexa 594-conjugated anti-mouse (Molecular Probes; Invitrogen)
Mitochondria	Human autoimmune serum recognizing the 70 kDa E2 subunit of the pyruvate dehydrogenase complex b.	1:400	Alexa 594-conjugated anti-human (Molecular Probes; Invitrogen)
Lysosome	Human autoimmune serum recognizing lysosomal proteinase	1:400	Alexa 488-conjugated anti-human (Molecular Probes; Invitrogen)
COXII	Rabbit polyclonal anti-COXII (GeneText)	1:200	Alexa 488-conjugated anti-rabbit (Molecular Probes; Invitrogen)

## Data Availability

The original contributions presented in the study are included in the article/Supplementary Material. Further inquiries can be directed to the corresponding author.

## Supplementary Materials

The following supporting information can be downloaded at: https://www.mdpi.com/article/10.3390/pharmaceutics16081015/s1, Figure S1. Clonogenic assay showing the time–response effect in control (a) condition and after 12 h (b) and 48 h (c) continuous treatment (CT) to 10 µM of Pt(IV)Ac-POA in MSTO-211H cell lines. Magnification: 4×. Figure S2. Graph showing the effects of Pt(IV)Ac-POA on cell viability of human MSTO-211H cell line. Table S1. Table showing the concentration of Pt(IV)Ac-POA able to induce cell death, causing a significant decrease (about 50%—IC50) in different cancer cell lines. * literature data.
