# Supplementary material for: Unraveling Novel Strategies in Mesothelioma Treatments Using a Newly Synthetized Platinum(IV) Compound
Source: Pharmaceutics. 2024 Jul 31;16(8):1015. doi: 10.3390/pharmaceutics16081015 (PMC11359418; doi:10.3390/pharmaceutics16081015)
Supplement: Supplementary file 1 [file pharmaceutics-16-01015-s001.zip › pharmaceutics-3082623-supplementary.pdf]

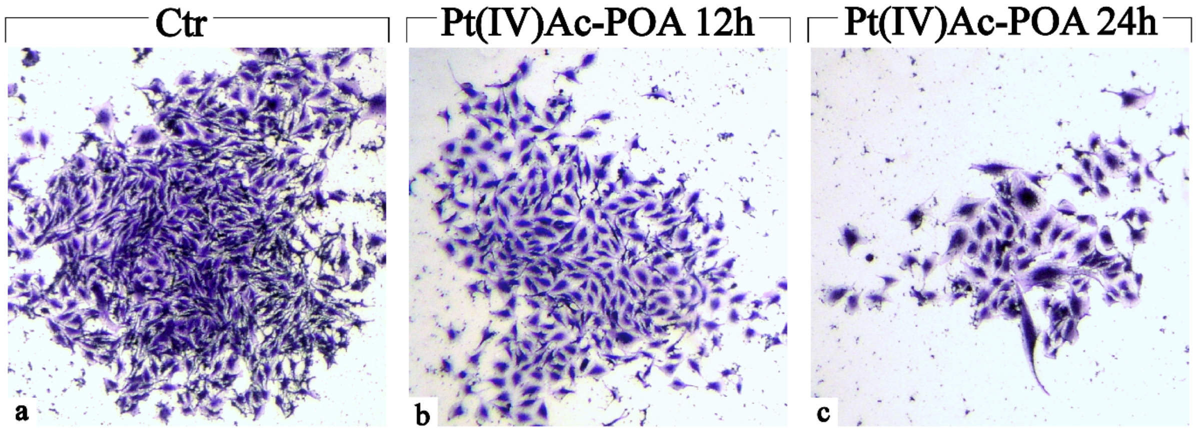

Figure S1. Clonogenic assay showing the time–response effect in control (**a**) condition and after 12h (**b**) and 48h (**c**) continuous treatment (CT) to 10μM of Pt(IV)Ac-POA in MSTO-211H cell lines. Magnification: 4x.

Cancer Cell Lines	IC50
MSTO	*9 μM*
U251 *	*10 μM*

Table S1. Table showing the concentration of Pt(IV)Ac-POA able to induce cell death, causing a significant decrease (about 50% - IC50) in different cancer cell lines. * literature data [29,30,42].

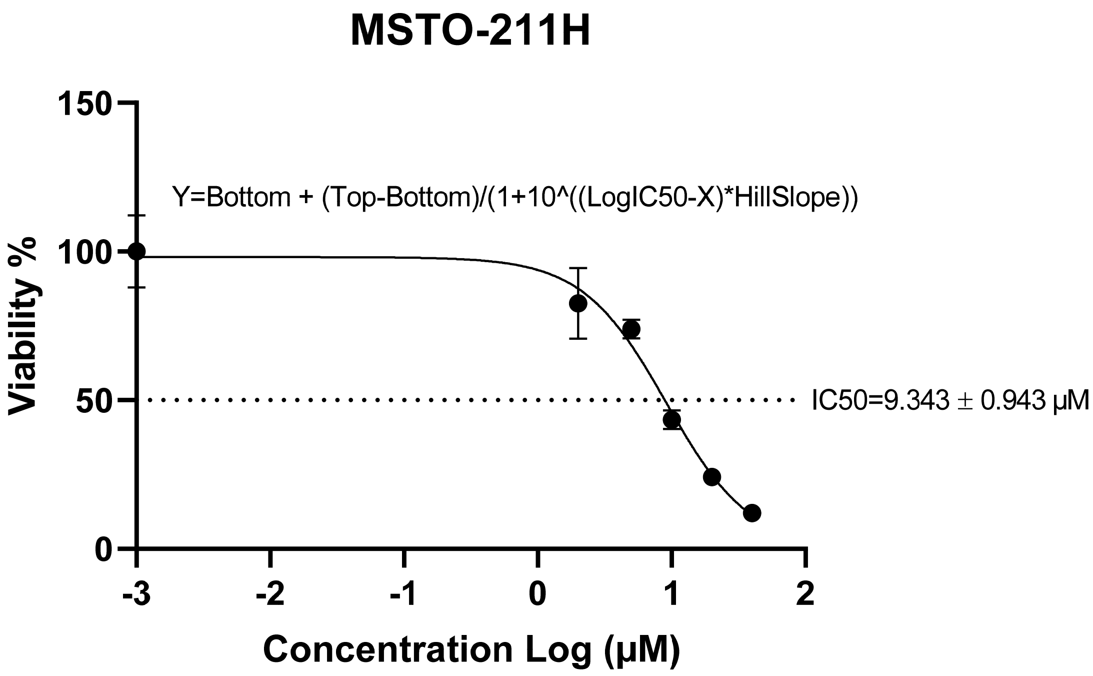

Figure S2. Graph showing the effects of Pt(IV)Ac-POA on cell viability of human MSTO-211H cell line.
